# Exudative Age-Related Macular Degeneration: Association between Treatment Efficacy and Single-Nucleotide Variants in *RAD51B*, *TRIB1*, *COL8A1*, *COL10A1*, *IL-9*, *IL-10*, and *VEGFA* Genes

**DOI:** 10.3390/ijms25136859

**Published:** 2024-06-22

**Authors:** Alvita Vilkeviciute, Dzastina Cebatoriene, Loresa Kriauciuniene, Dalia Zaliuniene, Rasa Liutkeviciene

**Affiliations:** 1 Neuroscience Institute, Lithuanian University of Health Sciences, Medical Academy, Eiveniu St. 2, LT-50161 Kaunas, Lithuania; loresa.kriauciuniene@lsmu.lt (L.K.); rasa.liutkeviciene@lsmu.lt (R.L.); 2 Medical Academy, Lithuanian University of Health Sciences, A. Mickeviciaus St. 9, LT-44307 Kaunas, Lithuania; dzastina.cebatoriene@lsmu.lt; 3 Department of Ophthalmology, Medical Academy, Lithuanian University of Health Sciences, Eiveniu St. 2, LT-50161 Kaunas, Lithuania; dalia.zaliuniene@lsmu.lt

**Keywords:** age-related macular degeneration, single-nucleotide variants, ELISA, anti-VEGFA therapy

## Abstract

Age-related macular degeneration (AMD) is a progressive neurodegenerative condition leading to vision loss and eventual blindness, with exudative AMD posing a heightened risk due to choroidal neovascularization and localized edema. Therapies targeting the VEGF pathway aim to address this mechanism for treatment effectiveness. Our study aimed to evaluate associations between specific genetic variants (*RAD51B* rs8017304, rs2588809; *TRIB1* rs6987702, rs4351379; *COL8A1* rs13095226; *COL10A1* rs1064583; *IL-9* rs1859430, rs2069870, rs11741137, rs2069885, rs2069884; *IL-10* rs1800871, rs1800872, rs1800896; *VEGFA* rs1570360, rs699947, rs3025033, rs2146323) and the response to anti-VEGF treatment for exudative AMD. We enrolled 119 patients with exudative AMD categorized as responders or non-responders based on their response to anti-VEGF treatment. Statistical analysis revealed that *RAD51B* rs8017304 heterozygous and homozygous minor allele carriers had increased CMT before treatment compared to wild-type genotype carriers (*p* = 0.004). Additionally, *TRIB1* rs4351379 heterozygous and homozygous minor allele carriers exhibited a greater decrease in central macular thickness (CMT) after 6 months of treatment than wild-type genotype carriers (*p* = 0.030). *IL-9* rs1859430, rs2069870, and rs2069884 heterozygous and homozygous minor allele carriers had worse BCVA before treatment than wild-type genotype carriers (*p* = 0.018, *p* = 0.012, *p* = 0.041, respectively). Conversely, *IL-9* rs2069885 heterozygous and homozygous minor allele carriers showed greater improvement in BCVA after 6 months compared to wild-type genotype carriers (*p* = 0.032). Furthermore, *VEGFA* rs699947 heterozygous and homozygous minor allele carriers had better BCVA before treatment and after 3 and 6 months of treatment than wild-type genotype carriers (*p* = 0.003, *p* = 0.022, respectively), with these carriers also exhibiting higher CMT after 6 months of anti-VEGF treatment (*p* = 0.032). Not all results remained statistically significant under this stringent correction for multiple comparisons. The comparisons of the serum concentrations of IL-10, VEGF-A, and VEGF-R2/KDR between non-responders and responders did not yield statistically significant differences. Our study identified significant associations between genetic variants, including *RAD51B* rs8017304, *TRIB1* rs4351379, *IL-9* rs1859430, rs2069870, rs2069884, rs2069885, and *VEGFA* rs699947, and parameters related to the efficacy of exudative AMD treatment, such as BCVA and CMT.

## 1. Introduction

Age-related macular degeneration (AMD) is a progressive neurodegenerative condition affecting the macula, resulting in vision loss and eventual blindness. The macula houses crucial photoreceptors for visual acuity and color perception [1]. Although typically diagnosed in individuals aged 60 and older [2], symptoms can appear as early as age 40 [3], leading to reduced visual acuity and quality of life, impacting activities such as facial recognition and color discrimination [4]. AMD comprises early, intermediate, and advanced stages, with exudative AMD carrying a higher risk of blindness [5,6]. Age, especially in developed nations with longer life expectancies, is closely associated with the prevalence of AMD [7], and projections suggest a substantial increase in cases by 2040 [8].

Crucial mechanisms involved in age-related changes in the eye, such as the formation of drusen and oxidative stress, play a significant role in AMD [9]. Drusen, coupled with reactive oxygen species (ROS), accumulate between the retinal pigment epithelium (RPE) and Bruch’s membrane (BrM), triggering chronic inflammation and causing damage to RPE cells and photoreceptors [10,11,12]. This process leads to the breakdown of the blood–ocular barrier, releasing inflammatory mediators and contributing to degeneration [13,14]. Disrupted inflammatory responses prompt an excessive release of proangiogenic factors like vascular endothelial factor A (VEGF-A), which fosters angiogenesis [15,16,17]. Exudative AMD, characterized by choroidal neovascularization, results in localized edema [17]. Therapies targeting the VEGF pathway aim to address this mechanism for treatment effectiveness [18].

Proteins play crucial roles in biological processes and are linked to many diseases, including age-related conditions. Recently, one of the most comprehensive studies of serum protein levels in various forms of AMD was conducted among a large elderly group, analyzing 4782 human serum proteins in relation to all genetic risk loci for AMD. The study found that serum proteins can indicate the severity of AMD independently of genetic factors and can predict the progression from early to advanced AMD. Additionally, several proteins were identified as causally related to AMD, with findings aligning with observational estimates [19]. Inflammatory mediators like IL-10 and IL-9, under the regulation of the NF-kB and JAK/STAT pathways, play a role in AMD development and have been identified as treatment targets [20]. Lifestyle elements such as smoking, alcohol intake, and inadequate diet contribute to AMD susceptibility, with genetics being the predominant factor, responsible for approximately 70% of cases [21]. These discoveries prompt us to explore potential biomarkers associated with early AMD diagnosis and treatment approaches.

Since genetic factors represent the primary risk contributors for AMD, we aimed to investigate the correlations between single-nucleotide variants (SNVs) in *IL-9* and IL-10, collagen-coding genes *COL8A1* and *COL10A1*, lipid transport-associated gene *TRIB*, oxidative stress and DNA damage-linked gene *RAD51B*, and the progressive angiogenesis-associated gene *VEGFA* concerning the effectiveness of exudative AMD treatment. We hypothesize that the selected SNVs and serum levels of IL-10, VEGF-A, and VEGF-R2/KDR may play a significant role in the response to anti-VEGFA treatment for exudative AMD.

## 2. Results

### 2.1. Associations between Central Macular Thickness and Best-Corrected Visual Acuity with Treatment Response

Our study group comprised 119 patients diagnosed with exudative AMD. We assessed the treatment response of exudative AMD and categorized patients into responders (*n* = 22) and non-responders (*n* = 97). We compared best-corrected visual acuity (BCVA) and central macular thickness (CMT) before treatment and after 3 and 6 months, as well as the changes in BCVA and CMT after 3 and 6 months, between responders and non-responders (Table 1).

Our findings revealed that after 6 months of treatment, responders exhibited significantly lower central macular thickness (CMT) compared to non-responders (median (IQR): 271 (96) vs. 319.5 (101), respectively, *p* = 0.034), along with better best-corrected visual acuity (BCVA) than non-responders (median (IQR): 0.40 (0.35) vs. 0.30 (0.29), respectively, *p* = 0.028). Additionally, there were significant differences in CMT and BCVA changes after 3 and 6 months of treatment between responders and non-responders. Specifically, CMT decreased significantly in responders compared to non-responders after 3 and 6 months (*p* = 0.005 and *p* = 0.001, respectively) (Table 1). BCVA increased more in responders compared to non-responders after 3 months (*p* = 0.003); however, after 6 months of treatment, BCVA increased more in non-responders compared to responders (*p* < 0.001) (Table 1).

Macular edema was assessed for all patients before treatment, but additional data were not recorded for all patients throughout the study. The results indicate that after 3 months of treatment, significantly fewer patients in the responder group had macular edema compared to the non-responders (*p* = 0.004). After six months of treatment, the edema decreased slightly in the non-responder group as well (Table 1).

### 2.2. Associations between Single-Nucleotide Variants (SNVs) and the Efficacy of Exudative AMD Treatment

The study includes 18 SNVs which were analyzed in our previous studies. Analysis showed associations between SNVs and early or exudative AMD development, so further statistical analysis let us evaluate the association between these SNVs and exudative AMD treatment efficacy.

### 2.3. RAD51B (rs8017304 and rs2588809), TRIB1 (rs6987702 and rs4351379), COL8A1 (rs13095226), and COL10A1 (rs1064583) Genetic Variant Associations with Exudative AMD Treatment Efficacy

At this stage of our study, we compared the distributions of *RAD51B* (rs8017304 and rs2588809), *TRIB1* (rs6987702 and rs4351379), *COL8A1* (rs13095226), and *COL10A1* (rs1064583) genotypes and alleles between responders and non-responders’ groups. However, the analysis did not reveal any statistically significant differences (Supplementary Material, Table S1).

Moreover, we wanted to find if there are associations between *RAD51B* (rs8017304 and rs2588809), *TRIB1* (rs6987702 and rs4351379), *COL8A1* (rs13095226), and *COL10A1* (rs1064583) and CMT or BCVA before anti-VEGF treatment and during the treatment. Statistical analysis showed that rs8017304 heterozygous and homozygous minor allele carriers had higher CMT before treatment than wild-type genotype carriers (*p* = 0.004) (Table 2). Also, we revealed that CMT decreased more for rs4351379 heterozygous and homozygous minor allele carriers than for wild-type genotype carriers after 6 months of treatment (*p* = 0.030), but these results did not survive the strict Bonferroni correction for multiple comparison. Any other associations between these SNVs and BCVA were determined (Table 3). Overall, these results show that *RAD51B* rs8017304 is associated with increased CMT in exudative AMD before treatment, but it is not associated with treatment efficacy. On the other hand, *TRIB1* rs4351379 is not associated with CMT in exudative AMD but with better anti-VEGF treatment response after 6 months (Table 3).

### 2.4. IL-9 and IL-10 Genetic Variant and IL-9 and IL-10 Serum Level Associations with Exudative AMD Treatment Efficacy

In this stage of the study, we compared the distributions of *IL-9* (rs1859430, rs2069870, rs11741137, rs2069885, rs2069884) and *IL-10* (rs1800871, rs1800872, and rs1800896) genotypes and alleles between responder and non-responder groups, but the analysis did not show any statistically significant differences (Supplementary Material, Table S2).

Also, we evaluated *IL-9* (rs1859430, rs2069870, rs11741137, rs2069885, rs2069884) and *IL-10* (rs1800871, rs1800872, and rs1800896) associations with CMT and BCVA before the treatment and during the treatment. Statistical analysis showed that rs1859430, rs2069870, and rs2069884 heterozygous and homozygous minor allele carriers had worse BCVA before treatment than wild-type genotype carriers (*p* = 0.018; *p* = 0.012; *p* = 0.041, respectively) (Table 3). The same Bonferroni correction for multiple comparison was applied, and it showed that the results did not remain significant after this correction. Moreover, we found that rs2069885 heterozygous and homozygous minor allele carriers had more improved BCVA after 6 months than wild-type genotype carriers (*p* = 0.032) (Table 3). These associations showed that *IL-9* rs1859430, rs2069870, and rs2069884 SNVs are associated with worse BCVA before anti-VEGF treatment, while rs2069885 might be associated with improved BCVA after 6 months of anti-VEGF treatment (Table 3).

IL-10 serum protein concentrations were measured compared between responders and non-responders, but the analysis did not reveal significant differences. (Supplementary Material Figure S1).

### 2.5. VEGFA Genetic Variant and VEGF-A and VEGF-R2/KDR Serum Level Associations with Exudative AMD Treatment Efficacy

Further analysis included SNVs at the *VEGFA* gene and VEGF-A and VEGF-R2/KDR serum protein level associations with exudative AMD treatment efficacy. We compared the distributions of *VEGFA* rs1570360, rs699947, rs3025033, and rs2146323 genotypes and alleles between responders and non-responders’ groups, but the analysis did not show any statistically significant differences either (Supplementary Material, Table S3).

In *VEGFA* SNVs and anti-VEGF treatment response analysis, we found that rs699947 heterozygous and homozygous minor allele carriers had better BCVA before treatment and after 3 and 6 months of treatment than wild-type genotype carriers (*p* = 0.027; *p* = 0.003; *p* = 0.022, respectively). Because of the Bonferroni correction, statistical significance was maintained only in the analysis of the genotype and BCVA results after 3 months of treatment.

Also, we found that rs699947 heterozygous and homozygous minor allele carriers had higher CMT after 6 months of anti-VEGF treatment than wild-type genotype carriers (*p* = 0.032) (Table 4). However, the results did not remain statistically significant after the Bonferroni correction was applied.

We additionally conducted comparisons of the serum concentrations of VEGF-A and VEGF-R2/KDR between non-responders and responders. Regrettably, our analysis did not reveal statistically significant differences between these groups (Supplementary Material Figures S2 and S3).

## 3. Discussion

Immunogenetic marker association analysis with exudative AMD treatment efficacy was performed in our study. We evaluated the exudative AMD treatment response and divided patients into responders (*n* = 22) and non-responders (*n* = 97) based on ophthalmological parameters.

Initially, we examined the distributions of *RAD51B* (rs8017304 and rs2588809), *TRIB1* (rs6987702 and rs4351379), *COL8A1* (rs13095226), and *COL10A1* (rs1064583) genotypes and alleles among responders and non-responders, yet no statistically significant differences were observed. However, we did find an association between *RAD51B* rs8017304 and increased central macular thickness (CMT) in exudative AMD, although it was not linked to treatment efficacy. Conversely, *TRIB1* rs4351379 showed no association with CMT in exudative AMD but exhibited a favorable response to anti-VEGF treatment after 6 months.

Further analysis revealed that *IL-9* rs1859430, rs2069870, and rs2069884 SNVs are associated with worse BCVA before anti-VEGF treatment. In contrast, rs2069885 might be associated with the improved BCVA after 6 months of anti-VEGF treatment.

The analysis of the *VEGFA* SNV revealed that the rs699947 C allele correlated with improved best-corrected visual acuity (BCVA) both before and after 3 and 6 months of treatment. Additionally, this SNV was linked to higher central macular thickness (CMT) after 6 months of anti-VEGF therapy, indicating that the *VEGFA* rs699947 C allele is associated with poorer treatment efficacy. To account for the potential type I error from multiple comparisons in our study, we applied the Bonferroni correction. Consequently, not all results remained statistically significant under this stringent correction.

Other researchers are also investigating the associations between genetic markers and the response to anti-VEGF treatment. They are particularly focusing on genetic variants previously linked to the development of exudative AMD, as these variants may impact the response to treatment with anti-VEGF agents. Well-studied genetic markers such as *HTRA1* (rs11200638), A69S at *LOC387715/ARMS2*, rs10490924 in *ARMS2/HTRA1*, or *CFH* Y402H have been associated with poorer visual outcomes following anti-VEGF treatment [22,23,24]. These findings suggest the potential for personalized treatment approaches based on patients’ genotypes to achieve optimal treatment responses in AMD.

While previous studies have not examined the association of *RAD51B* (rs8017304 and rs2588809), *TRIB1* (rs6987702 and rs4351379), *COL8A1* (rs13095226), *COL10A1* (rs1064583), *IL-9* (rs1859430, rs2069870, rs11741137, rs2069885, rs2069884), and *IL-10* (rs1800871, rs1800872, rs1800896) genetic variants with the response to exudative AMD treatment, *VEGFA* SNVs have been the focus of several studies as potential biomarkers. These SNVs could potentially aid in achieving optimal treatment responses and in developing individualized therapeutic approaches for exudative AMD.

Researchers conducted an analysis of the associations of rs699947 with anti-VEGF treatment and obtained similar findings. They observed that the rs699947 AA genotype was associated with a higher likelihood of improving best-corrected visual acuity or achieving a better overall response when intravitreal ranibizumab [25,26] or bevacizumab [27] was administered for AMD treatment. Additionally, *VEGFA* rs699947 was linked to better response to photodynamic therapy, not only with intravitreal injections [28]. Furthermore, several studies identified associations between *VEGFA* rs699947 and treatment efficacy: carriers of the AA genotype had a higher likelihood of a good response compared to other genotypes [29] or showed an association with retinal thickness [30], although statistical significance did not persist after corrections for multiple testing. However, one study reported no associations between *VEGFA* rs699947 and anti-VEGF response [31]. Conversely, conflicting results were reported in another study, where ranibizumab treatment was significantly more effective in patients carrying the C allele at rs699947, with the AA genotype associated with the absence of an early functional response to ranibizumab [32].

*VEGFA* rs1570360 and rs3025033 with two other *VEGFA* SNVs (rs699947 and rs2010963) were assessed for their association with the response to photodynamic therapy. However, no associations were detected [28,33].

Although the study by Yildiz et al. confirmed that *VEGFA* rs2146323 has no influence on the response to anti-VEGF treatment [31], discrepancies in rs2146323 genotype distributions were observed between PDT non-responders and PDT responders [28]. Hagstrom et al. illustrated an association between *VEGFA* rs2146323 and retinal thickness; however, the adjusted *p*-value did not reach statistical significance, indicating no associations with the response to anti-VEGF therapy (ranibizumab or bevacizumab) for exudative AMD [32].

Additional SNVs within the *VEGFA* gene (rs699946 and rs3025000) were similarly associated with improved visual outcomes following anti-VEGF treatment [34,35].

However, investigations into VEGF-A serum levels in response to intravitreal anti-VEGF treatment have been relatively limited. It has been demonstrated that aflibercept significantly decreases serum and plasma VEGF-A concentrations one month after injection [36], while conbercept notably reduces serum VEGF-A levels one day and one week after injection [37]. In contrast, ranibizumab has shown no significant effect on changes in serum or plasma VEGFA concentrations [39,40. In our study, we compared serum protein concentrations of VEGF-A and VEGF-R2/KDR between responders and non-responders; however, the analysis did not uncover significant differences. Notably, serum levels of VEGF-R2/KDR have not been previously analyzed in studies examining the response to exudative AMD treatment.

## 4. Materials and Methods

### 4.1. Ethics

This Lithuanian population-based case–control study was conducted according to the guidelines of the Declaration of Helsinki, and the protocol was approved by Kaunas Regional Biomedical Research Ethics Committee, Lithuanian University of Health Sciences (No. BE-2-/48). All participants were informed about the study and signed the informed consent form.

### 4.2. Study Design and Structure

#### Study Design

The study groups consisted of subjects who were admitted for the ophthalmological evaluation to the Ophthalmology Department, Hospital of Lithuanian University of Health Sciences, during the period from 2014 to 2023. An ophthalmological evaluation was performed for all the study subjects, and data about general health and other diseases were obtained during an examination by a family doctor and gathered from medical records.

### 4.3. Ophthalmological Evaluation

The present study subjects were evaluated by slit-lamp biomicroscope as described in our previous publication [21]. All AMD patients underwent optical coherence tomography (OCT), and optical coherence tomography angiography (OCT-A) was performed to confirm advanced AMD after the OCT examination.

### 4.4. AMD Group

**Figure 1:**
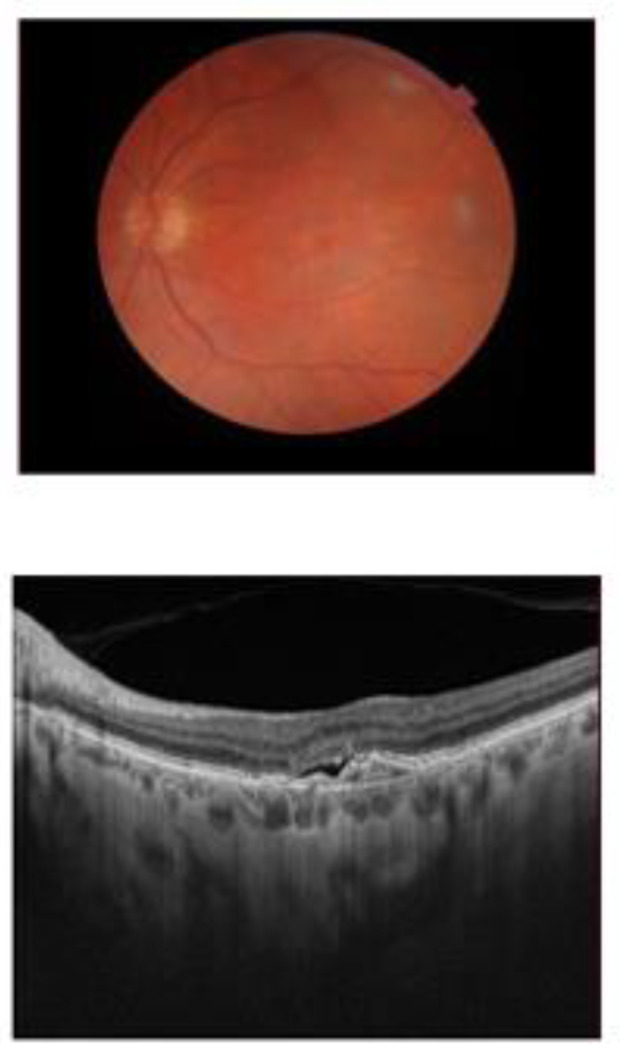
Macular changes in exudative AMD.

The AMD group consisted of subjects who underwent ophthalmological evaluation and were diagnosed with exudative AMD (Figure 1).

#### 4.4.1. AMD Exclusion Criteria

1. Unrelated eye disorders, e.g., high refractive error, cloudy cornea, lens opacity (nuclear, cortical, or posterior subcapsular cataract) except minor opacities, keratitis, acute or chronic uveitis, glaucoma, or diseases of the optic nerve;
2. Systemic illnesses, e.g., diabetes mellitus, malignant tumors, systemic connective tissue disorders, chronic infectious and non-infectious diseases, hypertension, coronary artery disease, stroke, or conditions following organ or tissue transplantation;
3. Ungraded color fundus photographs resulting from obscuring the ocular optic system or because of the low fundus photograph quality;
4. The use of antiepileptic or sedative drugs.

#### 4.4.2. Exudative AMD Response to Anti-VEGF Injection Treatment

The anti-VEGF treatment efficacy was evaluated for exudative AMD patients presenting exudative or hemorrhagic features in the macula but who had no previous intravitreal anti-VEGFA injections or any other treatment and were followed-up at least six months after the first injection of anti-VEGF. Central macular thickness (CMT) and best-corrected visual acuity (BCVA) measurements were performed before therapy, at three months, and at six months after the first anti-VEGF intravitreal injection.

Best-corrected visual acuity (BCVA) was assessed using a Snellen chart before treatment and six months after the first intravitreal anti-VEGF injection. A deterioration in visual acuity was defined as a loss of one or more lines (>5 letters) on the chart. Changes in BCVA during the treatment period were calculated using the following formula: BCVA after six months minus BCVA before treatment.

A good response was defined as the resolution of fluid according to OCT six months after the first injection and/or an improvement of >5 letters. Non-response was defined as an increase in fluid of 100 μM (IRF, SRF, and CMT) or increasing hemorrhage compared to baseline and/or a loss of >5 letters compared to baseline or best-corrected vision subsequently. Changes in CMT were calculated as follows: CMT before treatment minus CMT after six months.

### 4.5. Control Group

Subjects who underwent ophthalmological evaluation were involved in the control group.

#### 4.5.1. Control Group Inclusion Criteria

1. Older than 18 years;
2. Patients after senile cataract surgeries (without any other ocular comorbidities);
3. Signed informed consent form.

#### 4.5.2. Control Group Exclusion Criteria

1. Unrelated eye disorders, e.g., high refractive error, cloudy cornea, lens opacity (nuclear, cortical, or posterior subcapsular cataract) except minor opacities, keratitis, acute or chronic uveitis, glaucoma, or diseases of the optic nerve;
2. Systemic illnesses, e.g., diabetes mellitus, malignant tumors, systemic connective tissue disorders, chronic infectious and non-infectious diseases, hypertension, coronary artery disease, stroke, or conditions following organ or tissue transplantation;
3. Ungraded color fundus photographs resulting from obscuring the ocular optic system or because of fundus photograph quality;
4. The use of antiepileptic or sedative drugs.

### 4.6. Deoxyribonucleic Acid Extraction from Peripheral Venous Blood and Genotyping

Deoxyribonucleic acid (DNA) extraction and the genotyping of eighteen single-nucleotide variants (SNVs) were conducted, targeting *RAD51B* (rs8017304 and rs2588809), *TRIB1* (rs6987702 and rs4351379), *COL8A1* (rs13095226), *COL10A1* (rs1064583), *IL-9* (rs1859430, rs2069870, rs11741137, rs2069885, and rs2069884), *IL-10* (rs1800871, rs1800872, and rs1800896), and *VEGFA* (rs1570360, rs699947, rs3025033, and rs2146323). These procedures were performed at the Laboratory of Ophthalmology, Neuroscience Institute, Lithuanian University of Health Sciences, utilizing predesigned TaqManTM Genotyping assays from Thermo Fisher Scientific, Pleasanton, CA, USA, following the manufacturer’s guidelines and as previously described in our publications [38,39,40].

### 4.7. Single-Nucleotide Variant Selection

The selection of single-nucleotide variants (SNVs) for this study was based on previously published research and established associations with AMD and related biological pathways, specifically as follows:

1. ***RAD51B, TRIB1, COL8A1*, and *COL10A1 g*enetic variants**: These variants were chosen due to their direct associations with AMD development, their involvement in processes such as oxidative stress and DNA damage linked to aging and AMD, and their regulation by inflammatory stimulation. Additionally, their associations with AMD in other populations were considered.
2. ***IL-9* and *IL-10* genetic variants**: These variants were selected based on interactions between IL-9 and IL-10, their minor allele frequencies, and previous associations with diseases like asthma and allergic rhinitis. This study marks their first use as potential biomarkers for AMD.
3. ***VEGFA* genetic variants**: These variants were chosen based on haplotype block coverage and prior research on VEGFA variants, which have shown inconsistent results regarding AMD associations.

These SNVs were identified and chosen for their potential roles as biomarkers in the development and progression of early and exudative AMD. More accurate SNP selection criteria were described in our previous publications [21,38,39].

### 4.8. Serum Concentration Measurement

The obtained serum was aliquoted into 200 μL portions in Eppendorf tubes and stored at −80 °C. Human IL-9, IL-10, VEGF-A, and VEGF-R2/KDR assays were conducted using the Invitrogen ELISA Kit. For human IL-9 (Cat. No. BMS2081, Thermofisher Scientific, Waltham, MA, USA), the assay range was 3.1–200 pg/mL with a sensitivity of 0.5 pg/mL. For human IL-10 (Cat. No. BMS215-2, Thermofisher Scientific, Waltham, MA, USA), the assay range was 3.15–200 pg/mL with a sensitivity of 1 pg/mL. For VEGF-A (Cat. No. BMS277-2, Thermofisher Scientific, Waltham, MA, USA), the assay range was 15.6–1000 pg/mL with a sensitivity of 7.9 pg/mL. For VEGF-R2/KDR (Cat. No. BMS2019, Thermofisher Scientific, Waltham, MA, USA), the assay range was 78–5000 pg/mL with a sensitivity of 7 pg/mL, all following the manufacturer’s instructions. Protein concentrations were determined and calculated using the Multiskan FC Microplate Photometer (Thermo Scientific, Waltham, MA, USA) at 450 nm. Samples were excluded if serum cytokine concentrations fell below the detection range.

### 4.9. Statistical Analysis

Statistical analysis was performed using the SPSS/W 27.0 software (Statistical Package for the Social Sciences for Windows, Inc., Chicago, IL, USA). Continuous data (age, protein serum concentrations, BCVA, and CMT) were evaluated for normality by the Shapiro–Wilk test. Continuous variables presented the median with an interquartile range (IQR) based on non-normal data distributions. The Mann–Whitney test was used to compare two groups for non-normally distributed data.

Categorical data (gender and genotype distributions) are presented as absolute numbers with percentages in brackets and compared between groups using the *Chi-*square** (χ2) test. Fisher’s exact test was used only to compare the allele distributions between exudative AMD subgroups, ‘responders’ and ‘non-responders’, when the number of subjects was less than 50 in one subgroup.

Due to the multiple association calculations, we introduced a Bonferroni correction and applied an adjusted significance threshold for multiple comparisons α = 0.0028 (0.05/18, as we analyzed eighteen different SNVs).

Graphs were created using GraphPad Prism version 9.0.0 for Mac (GraphPad Software, San Diego, CA, USA).

## 5. Conclusions

We identified significant associations between genetic variants, including *RAD51B* rs8017304, *TRIB1* rs4351379, *IL-9* rs1859430, rs2069870, rs2069884, rs2069885, and *VEGFA* rs699947, and parameters related to the efficacy of exudative AMD treatment, such as BCVA and CMT. While our study yielded significant results, we recommend that all these SNVs be considered in future research endeavors aimed at identifying optimal treatment responses for exudative AMD or treatments for other forms of AMD.

## Figures and Tables

**Table 1:** The relationship between central macular thickness and best-corrected visual acuity and their association with the response to anti-VEGF treatment in patients with exudative AMD.

Parameter	Measurement Point	Responder Median (IQR) *n* = 97	Non-Responder Median (IQR) *n* = 22	*p* Value
CMT (μM)	Before treatment	338 (100.0)	284 (108.5)	0.269
	After 3 months	270 (100)	291 (107.5)	0.315
	After 6 months	271 (96)	319.5 (101)	**0.034**
CMT changes (μM)	After 3 months	45 (98)	10 (51)	**0.005**
	After 6 months	51 (105)	−10 (144)	**0.001**
BCVA	Before treatment	0.30 (0.24)	0.40 (0.42)	0.306
	After 3 months	0.40 (0.30)	0.30 (0.45)	0.607
	After 6 months	0.40 (0.35)	0.30 (0.29)	**0.028**
BCVA changes	After 3 months	0.025 (0.10)	−0.1 (0.09)	**0.003**
	After 6 months	0.05 (0.13)	0.30 (0.29)	**<0.001**
Macular edema, *n* (%)	Before treatment			-
	Fluid	91 (100)	22 (100)	
	No fluid	0	0	
	After 3 months			**0.004**
	Fluid	53 (58.2)	20 (90.9)	
	No fluid	38 (41.8)	2 (9.1)	
	After 6 months			0.225
	Fluid	30 (66.7)	14 (82.4)	
	No fluid	15 (33.3)	3 (17.6)	

BCVA—best-corrected visual acuity; CMT—central macular thickness; *p*-significance level, statistically significant when *p* < 0.05; *p*-values marked with bold indicate statistically significant *p*-values.

**Table 2:** Associations between the distribution of *RAD51B* (rs8017304 and rs2588809), *TRIB1* (rs6987702 and rs4351379), *COL8A1* (rs13095226), and *COL10A1* (rs1064583) and central macular thickness and best-corrected visual acuity.

Parameter	Measurement Point	Median (IQR)	Median (IQR)	*p* Value
*RAD51B* rs8017304				
		AG + GG	AA	
CMT (μM)	Before treatment	353 (57)	303.5 (97.75)	**0.004**
	After 3 months	320 (114.5)	278 (97.25)	0.051
	After 6 months	297 (113)	281 (83.5)	0.205
CMT changes (μM)	After 3 months	21.5 (77.75)	19 (62.5)	0.831
	After 6 months	26 (100.5)	25 (80)	0.488
BCVA	Before treatment	0.3 (0.3)	0.2 (0.25)	0.624
	After 3 months	0.3 (0.47)	0.275 (0.32)	0.580
	After 6 months	0.3 (0.38)	0.3 (0.25)	0.630
BCVA changes	After 3 months	0 (0.1)	0.045 (0.11)	0.911
	After 6 months	0 (0.14)	0.045 (0.1)	0.464
*RAD51B* rs2588809				
		CT + TT	CC	
CMT (μM)	Before treatment	350 (112)	329 (73)	0.217
	After 3 months	270 (86)	307 (114)	0.951
	After 6 months	295 (76)	283 (108)	0.872
CMT changes (μM)	After 3 months	25 (86)	19 (61)	0.217
	After 6 months	30 (91)	39 (98)	0.576
BCVA	Before treatment	0.25 (0.24)	0.25 (0.24)	0.093
	After 3 months	0.25 (0.3)	0.32 (0.44)	0.175
	After 6 months	0.2 (0.32)	0.3 (0.34)	0.201
BCVA changes	After 3 months	0 (0.1)	0.03 (0.1)	0.841
	After 6 months	0 (0.16)	0.02 (0.1)	0.880
*TRIB1* rs6987702				
		TC + CC	TT	
CMT (μM)	Before treatment	349.5 (69.25)	326 (93.5)	0.090
	After 3 months	326.5 (124.5)	278 (183.25)	0.092
	After 6 months	306 (128.5)	282 (68.75)	0.457
CMT changes (μM)	After 3 months	15 (69.75)	20.5 (69.75)	0.562
	After 6 months	47 (154)	28.5 (70.75)	0.471
BCVA	Before treatment	0.275 (0.27)	0.25 (0.27)	0.645
	After 3 months	0.35 (0.47)	0.275 (0.35)	0.792
	After 6 months	0.31 (0.37)	0.3 (0.32)	0.510
BCVA changes	After 3 months	0.05 (0.21)	0 (0.1)	0.839
	After 6 months	0.005 (0.21)	0.025 (0.1)	0.641
*TRIB1* rs4351379				
		GC + CC	GG	
CMT (μM)	Before treatment	357 (138)	337 (76.5)	0.444
	After 3 months	253.5 (136.5)	307 (92.5)	0.294
	After 6 months	265.5 (86.25)	297 (101)	0.133
CMT changes (μM)	After 3 months	36.5 (144)	19 (62)	0.170
	After 6 months	87.5 (88.25)	26.5 (93.5)	**0.030**
BCVA	Before treatment	0.325 (0.21)	0.25 (0.25)	0.592
	After 3 months	0.45 (0.53)	0.275 (0.32)	0.497
	After 6 months	0.41 (0.37)	0.3 (0.32)	0.332
BCVA changes	After 3 months	0.125 (0.32)	0 (0.1)	0.591
	After 6 months	0.085 (0.16)	0 (0.16)	0.178
*COL8A1* rs13095226				
		TC + CC	TT	
CMT (μM)	Before treatment	326 (80)	347 (100)	0.795
	After 3 months	280 (64)	308 (127)	0.786
	After 6 months	282 (65)	288 (116)	0.762
CMT changes (μM)	After 3 months	19 (54)	22 (82)	0.879
	After 6 months	30 (72)	39 (126)	0.696
BCVA	Before treatment	0.2 (0.25)	0.3 (0.24)	0.733
	After 3 months	0.3 (0.35)	0.3 (0.34)	0.697
	After 6 months	0.3 (0.25)	0.32 (0.35)	0.495
BCVA changes	After 3 months	0 (0.2)	0.03 (0.1)	0.524
	After 6 months	0 (0.14)	0.04 (0.1)	0.170
*COL10A1* rs1064583				
		AG + GG	AA	
CMT (μM)	Before treatment	342 (114)	330.6 (61)	0.949
	After 3 months	307 (124.5)	285 (75.5)	0.578
	After 6 months	295 (130)	283 (86)	0.872
CMT changes (μM)	After 3 months	21 (71)	19 (71)	0.872
	After 6 months	30 (86)	39 (116)	0.921
BCVA	Before treatment	0.25 (0.25)	0.25 (0.25)	0.942
	After 3 months	0.3 (0.42)	0.3 (0.29)	0.698
	After 6 months	0.32 (0.35)	0.3 (0.23)	0.669
BCVA changes	After 3 months	0.04 (0.15)	0 (0.1)	0.455
	After 6 months	0.02 (0.13)	0 (0.1)	0.541

BCVA—best-corrected visual acuity; CMT—central macular thickness; *p*-significance level, statistically significant when *p* < 0.05; *p*-values marked with bold indicate statistically significant *p*-values.

**Table 3:** Associations between distribution of *IL-9* (rs1859430, rs2069870, rs11741137, rs2069885, rs2069884) and *IL-10* (rs1800871, rs1800872, and rs1800896) and central macular thickness and best-corrected visual acuity.

Parameter	Measurement Point	Median (IQR)	Median (IQR)	*p* Value
*IL-9* rs1859430				
		GA + AA	GG	
CMT (μM)	Before treatment	325 (104.5)	334.12 (99)	0.697
	After 3 months	264 (83.5)	287.63 (122.5)	0.729
	After 6 months	271 (87)	284.5 (110.75)	0.266
CMT changes (μM)	After 3 months	31 (73.5)	23.5 (89.5)	0.599
	After 6 months	26 (100.5)	30 (106.25)	0.845
BCVA	Before treatment	0.25 (0.25)	0.32 (0.3)	**0.018**
	After 3 months	0.3 (0.25)	0.4 (0.43)	0.124
	After 6 months	0.3 (0.26)	0.31 (0.45)	0.714
BCVA changes	After 3 months	0 (0.1)	0.035 (0.11)	0.750
	After 6 months	0.02 (0.1)	0 (0.2)	0.136
*IL-9* rs2069870				
		AG + GG	AA	
CMT (μM)	Before treatment	327.5 (102.75)	336 (104)	0.744
	After 3 months	265 (91)	276 (114.5)	0.911
	After 6 months	272 (89.5)	283 (105)	0.422
CMT changes (μM)	After 3 months	28 (74.75)	24 (88.5)	0.774
	After 6 months	25 (101)	30 (103)	0.623
BCVA	Before treatment	0.25 (0.25)	0.32 (0.3)	**0.012**
	After 3 months	0.3 (0.24)	0.4 (0.43)	0.097
	After 6 months	0.3 (0.25)	0.32 (0.45)	0.639
BCVA changes	After 3 months	0 (0.1)	0.03 (0.12)	0.703
	After 6 months	0.016 (0.1)	0 (0.2)	0.122
*IL-9* rs11741137				
		CT + TT	CC	
CMT (μM)	Before treatment	330 (103)	332.5 (119)	0.752
	After 3 months	264 (93)	276.5 (109.25)	0.977
	After 6 months	271 (55)	284.5 (116.5)	0.291
CMT changes (μM)	After 3 months	42 (86)	24 (84.75)	0.544
	After 6 months	33 (103)	28.5 (102.5)	0.564
BCVA	Before treatment	0.3 (0.25)	0.31 (0.3)	0.070
	After 3 months	0.4 (0.24)	0.335 (0.42)	0.324
	After 6 months	0.32 (0.3)	0.3 (0.42)	0.761
BCVA changes	After 3 months	0 (0.1)	0 (0.1)	0.313
	After 6 months	0.02 (0.1)	0 (0.2)	0.057
*IL-9* rs2069885				
		GA + AA	GG	
CMT (μM)	Before treatment	332 (109.5)	327.5 (113)	0.405
	After 3 months	264 (94.5)	276.5 (107.5)	0.657
	After 6 months	271 (66.5)	284.5 (116.25)	0.510
CMT changes (μM)	After 3 months	25 (83)	26 (86.25)	0.640
	After 6 months	24 (104)	30 (103.75)	0.547
BCVA	Before treatment	0.3 (0.25)	0.31 (0.3)	0.053
	After 3 months	0.4 (0.29)	0.35 (0.41)	0.261
	After 6 months	0.30 (0.32)	0.3 (0.38)	0.761
BCVA changes	After 3 months	0 (0.11)	0 (0.1)	0.348
	After 6 months	0.02 (0.11)	0 (0.2)	**0.032**
*IL-9* rs2069884				
		GT + TT	GG	
CMT (μM)	Before treatment	332.5 (109.5)	327.5 (113)	0.623
	After 3 months	264 (94.5)	276.5 (107.5)	0.824
	After 6 months	271 (66.5)	284.5 (116.25)	0.416
CMT changes (μM)	After 3 months	25 (83)	26 (86.25)	0.703
	After 6 months	24 (104)	30 (103.75)	0.669
BCVA	Before treatment	0.3 (0.25)	0.31 (0.3)	**0.041**
	After 3 months	0.4 (0.29)	0.35 (0.41)	0.258
	After 6 months	0.3 (0.32)	0.3 (0.38)	0.895
BCVA changes	After 3 months	0 (0.11)	0 (0.1)	0.266
	After 6 months	0.02 (0.11)	0 (0.2)	0.050
*IL-10* rs1800871				
		GA + AA	GG	
CMT (μM)	Before treatment	351.5 (108.5)	309 (83.5)	0.288
	After 3 months	276 (96)	262 (107.5)	0.451
	After 6 months	281 (92.5)	271 (106.5)	0.760
CMT changes (μM)	After 3 months	26.5 (100.25)	22 (75)	0.499
	After 6 months	36.5 (121)	25 (90)	0.393
BCVA	Before treatment	0.3 (0.27)	0.3 (0.2)	0.662
	After 3 months	0.375 (0.41)	0.4 (0.3)	0.466
	After 6 months	0.29 (0.38)	0.4 (0.28)	0.719
BCVA changes	After 3 months	0 (0.1)	0 (0.11)	0.776
	After 6 months	0.005 (0.18)	0 (0.12)	0.821
*IL-10* rs1800872				
		GT + TT	GG	
CMT (μM)	Before treatment	351.5 (108.5)	309 (83.5)	0.288
	After 3 months	276 (96)	262 (107.5)	0.451
	After 6 months	281 (92.5)	271 (106.5)	0.760
CMT changes (μM)	After 3 months	26.5 (100.25)	22 (75)	0.499
	After 6 months	36.5 (121)	25 (95)	0.393
BCVA	Before treatment	0.3 (0.27)	0.3 (0.2)	0.662
	After 3 months	0.375 (0.41)	0.4 (0.3)	0.466
	After 6 months	0.29 (0.38)	0.4 (0.28)	0.719
BCVA changes	After 3 months	0 (0.1)	0 (0.11)	0.776
	After 6 months	0.005 (0.18)	0 (0.12)	0.821
*IL-10* rs1800896				
		TC + CC	TT	
CMT (μM)	Before treatment	323 (117)	344 (79.5)	0.981
	After 3 months	276 (109)	270 (91)	0.947
	After 6 months	282 (106)	268 (90.25)	0.865
CMT changes (μM)	After 3 months	28 (87)	24 (80)	0.769
	After 6 months	27 (100)	34.5 (107.25)	0.936
BCVA	Before treatment	0.3 (0.34)	0.3 (0.23)	0.458
	After 3 months	0.32 (0.3)	0.4 (0.41)	0.107
	After 6 months	0.3 (0.35)	0.335 (0.35)	0.060
BCVA changes	After 3 months	0 (0.1)	0.04 (0.2)	0.103
	After 6 months	0 (0.17)	0.015 (0.17)	0.184

BCVA—best-corrected visual acuity; CMT—central macular thickness; *p*-significance level, statistically significant when *p* < 0.05; *p*-values marked with bold indicate statistically significant *p*-values.

**Table 4:** Associations between *VEGFA* rs1570360, rs699947, rs3025033, and rs2146323 and central macular thickness and best-corrected visual acuity.

Parameter	Measurement Point	Median (IQR)	Median (IQR)	*p* Value
*VEGFA* rs1570360				
		GA + AA	GG	
CMT (μM)	Before treatment	353.5 (64.75)	325 (107)	0.547
	After 3 months	318 (120.25)	270 (98)	0.732
	After 6 months	268 (86)	293 (123)	0.282
CMT changes (μM)	After 3 months	24 (89)	42 (109.5)	0.458
	After 6 months	55 (109)	27 (113)	0.347
BCVA	Before treatment	0.25 (0.35)	0.30 (0.24)	0.312
	After 3 months	0.375 (0.27)	0.32 (0.35)	0.570
	After 6 months	0.325 (0.37)	0.30 (0.32)	0.736
BCVA changes	After 3 months	0.035 (0.11)	0.00 (0.10)	0.364
	After 6 months	0.05 (0.27)	0.01 (0.10)	0.603
*VEGFA* rs699947				
		AC + CC	AA	
CMT (μM)	Before treatment	308.5 (122.5)	341 (95)	0.650
	After 3 months	273.5 (89.25)	276 (101)	0.358
	After 6 months	293 (104)	257 (50)	**0.032**
CMT changes (μM)	After 3 months	24 (108)	32 (73)	0.476
	After 6 months	27 (123.5)	58 (96)	0.227
BCVA	Before treatment	0.40 (0.21)	0.30 (0.25)	**0.027**
	After 3 months	0.40 (0.30)	0.30 (0.34)	**0.003**
	After 6 months	0.40 (0.30)	0.30 (0.35)	**0.022**
BCVA changes	After 3 months	0.00 (0.18)	0.00 (0.10)	0.508
	After 6 months	0.025 (0.18)	0.02 (0.10)	0.621
*VEGFA* rs3025033				
		AG + GG	AA	
CMT (μM)	Before treatment	266.5 (-)	332 (102.5)	0.562
	After 3 months	233 (-)	276 (102.5)	0.263
	After 6 months	282 (107)	282 (102)	0.763
CMT changes (μM)	After 3 months	15 (102)	39 (87)	0.502
	After 6 months	14 (123)	48 (98)	0.267
BCVA	Before treatment	0.16 (-)	0.30 (0.24)	0.510
	After 3 months	0.30 (-)	0.35 (0.35)	0.773
	After 6 months	0.275 (-)	0.32 (0.30)	0.700
BCVA changes	After 3 months	0.14 (-)	0.00 (0.10)	0.427
	After 6 months	0.115 (-)	0.02 (0.10)	0.195
*VEGFA* rs2146323				
		CA + AA	CC	
CMT (μM)	Before treatment	285 (73)	341.5 (100)	0.068
	After 3 months	262 (53)	277.5 (100.75)	0.367
	After 6 months	281 (85.5)	283 (119)	0.779
CMT changes (μM)	After 3 months	26.5 (79.25)	46.5 (130.5)	0.957
	After 6 months	34.5 (93.25)	40.5 (158.75)	0.709
BCVA	Before treatment	0.25 (0.35)	0.30 (0.24)	0.963
	After 3 months	0.40 (0.30)	0.335 (0.37)	0.835
	After 6 months	0.30 (0.40)	0.31 (0.30)	0.992
BCVA changes	After 3 months	0.05 (0.10)	0.00 (0.10)	0.496
	After 6 months	0.05 (0.15)	0.005 (0.10)	0.705

BCVA—best-corrected visual acuity; CMT—central macular thickness; *p*-significance level, statistically significant when *p* < 0.05; *p*-values marked with bold indicate statistically significant *p*-values.

## Data Availability

The datasets used and/or analyzed during the current study are available from the corresponding author on reasonable request.

## Supplementary Materials

The following supporting information can be downloaded at https://www.mdpi.com/article/10.3390/ijms25136859/s1.
