# Supplementary material for: Exudative Age-Related Macular Degeneration: Association between Treatment Efficacy and Single-Nucleotide Variants in RAD51B, TRIB1, COL8A1, COL10A1, IL-9, IL-10, and VEGFA Genes
Source: Int J Mol Sci. 2024 Jun 22;25(13):6859. doi: 10.3390/ijms25136859 (PMC11241034; doi:10.3390/ijms25136859)
Supplement: Supplementary file 1 [file ijms-25-06859-s001.zip › ijms-3043219-supplementary.pdf]

Supplementary material

Table S1. Distribution of *RAD51B* (rs8017304; rs2588809), *TRIB1* (rs6987702; rs4351379), *COL8A1* (rs13095226), *COL10A1* (rs1064583) genotypes and alleles in responders and non-responders

Gene/marker/Genotype/allele	*Responders n (%) (N=45)*	*Non-responders n (%) (N=12)*	*p value**
RAD51B rs8017304			
AA	26 (57.8)	4 (33.3)	
AG	17 (37.8)	6 (50)	0.179
GG	2 (4.2)	2 (16.7)	
A	69 (76.7)	14 (58.3)	0.119
G	21 (23.3)	10 (41.7)	
RAD51B rs2588809			
CC	36 (80)	8 (66.7)	
CT	9 (20)	4 (33.3)	0.269
TT	-	-	
C	81 (90)	20 (83.3)	0.468
T	9 (10)	4 (16.7)	
TRIB1 rs6987702			
TT	27 (60)	8 (66.7)	
TC	16 (35.6)	3 (25)	0.720
CC	2 (4.4)	1 (8.3)	
T	70 (77.8)	19 (79.2)	1
C	20 (22.2)	5 (20.8)	
TRIB1 rs4351379			
GG	39 (86.7)	11 (91.7)	
GC	6 (13.3)	1 (8.3)	0.541
CC	-	-	
G	84 (93.3)	23 (95.8)	1
C	6 (6.7)	1 (4.5)	
COL8A1 rs13095226			
TT	27 (60)	6 (50)	
TC	17 (37.8)	5 (41.7)	0.546
CC	1 (2.2)	1 (8.3)	
T	71 (78.9)	17 (70.8)	0.419
C	19 (21.1)	7 (29.2)	
COL10A1 rs1064583			
AA	19 (42.2)	6 (50)	
AG	22 (48.9)	5 (41.7)	0.888
GG	4 (8.9)	1 (8.3)	
A	60 (66.7)	17 (70.8)	0.809
G	30 (33.3)	7 (29.2)	

*p-significance level, p is statistically significant when <0.05; *Fisher Exact Test was used to compare the allele distributions.*

Table S2. Distribution of *IL-9* (rs1859430, rs2069870, rs11741137, rs2069885, rs2069884) and *IL-10* (rs1800871, rs1800872 and rs1800896) genotypes and alleles in responders and non-responders

Gene/marker/Genotype/allele	*Responders n (%) (N=97)*	*Non-responders n (%) (N=22)*	*p value**
IL-9 rs1859430			
GG	50 (51.5)	13 (59.1)	
GA	42 (43.3)	8 (36.4)	0.814
AA	5 (5.2)	1 (4.5)	
G	142 (73.2)	34 (77.3)	0.704
A	52 (26.3)	10 (22.7)	
IL-9 rs2069870			
AA	49 (50.5)	13 (59.1)	
AG	48 (49.5)	9 (40.9)	0.313
GG	-	-	
A	146 (75.3)	35 (79.5)	0.696
G	48 (24.7)	9 (20.5)	
IL-9 rs11741137			
CC	56 (57.7)	15 (68.2)	
CT	39 (40.2)	6 (27.3)	0.458
TT	2 (2.1)	1 (4.5)	
C	151 (77.8)	36 (81.8)	0.686
T	43 (22.2)	8 (18.2)	
IL-9 rs2069885			
GG	58 (59.8)	15 (68.2)	
GA	37 (38.1)	6 (27.3)	0.540
AA	2 (2.1)	1 (4.5)	
G	153 (78.9)	36 (81.8)	0.837
A	41 (21.1)	8 (18.2)	
IL-9 rs2069884			
GG	58 (59.8)	15 (68.2)	
GT	37 (38.1)	6 (27.3)	0.540
TT	2 (2.1)	1 (4.5)	
G	153 (78.9)	36 (81.8)	0.837
T	41 (21.1)	8 (18.2)	
IL-10 rs1800871			
GG	55 (56.7)	11 (50)	
GA	36 (37.1)	8 (36.4)	0.481
AA	6 (6.2)	3 (13.6)	
G	146 (75.3)	30 (68.2)	0.345
A	48 (24.7)	14 (31.8)	
IL-10 rs1800872			
GG	55 (56.7)	11 (50)	
GT	36 (37.1)	8 (36.4)	0.481
TT	6 (6.2)	3 (13.6)	
G	146 (75.3)	30 (68.2)	0.345

T	48 (24.7)	14 (31.8)	
IL-10 rs1800896			
TT	29 (29.9)	7 (31.8)	
TC	46 (47.4)	10 (45.5)	0.982
CC	22 (22.7)	5 (22.7)	
T	104 (53.6)	24 (54.5)	1
C	90 (46.4)	20 (45.5)	

*p-significance level, p is statistically significant when <0.05; *Fisher Exact Test was used to compare the allele distributions.*

Table S3. Distribution of *VEGFA* rs1570360, rs699947, rs3025033, rs2146323 genotypes and alleles in responders and non-responders

Gene marker/Genotype/allele	***Responders n (%) (N=97)***	***Non-responders n (%) (N=22)***	***p value****
VEGFA rs1570360			
GG	48 (48.5)	9 (40.9)	0.524
GA	36 (37.1)	11 (50.0)	
AA	13 (13.4)	2 (9.1)	
G	132 (68.0)	29 (65.9)	0.859
A	62 (32.0)	15 (34.1)	
VEGFA rs699947			
AA	27 (27.8)	4 (18.2)	
AC	36 (37.1)	14 (63.6)	0.073
CC	34 (35.1)	4 (18.2)	
A	90 (46.4)	22 (50.0)	0.739
C	104 (53.6)	22 (50.0)	
VEGFA rs3025033			
AA	71 (73.2)	14 (63.6)	0.233
AG	24 (24.7)	6 (27.3)	
GG	2 (2.1)	2 (9.1)	
A	166 (85.6)	34 (77.4)	0.177
G	28 (14.4)	10 (22.7)	
VEGFA rs2146323			
CC	49 (50.5)	10 (45.5)	0.811
CA	37 (38.1)	10 (45.5)	
AA	11 (11.3)	2 (9.1)	
C	135 (69.6)	30 (68.2)	0.858
A	59 (30.4)	14 (31.8)	

*p-significance level, statistically significant when p<0.05; p-values marked with bold indicate statistically significant p-values; *Fisher Exact Test was used for allele comparison.*

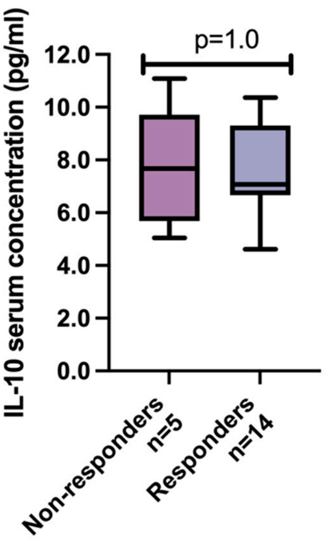

Figure S1. IL-10 serum concentrations between non-responders and responders.

The bars represent the median with interquartile range (1st quartile and 3rd quartile). VEGF-A serum concentration in non-responders' group: 7.67 (4.04) *pg/ml and responders' group:* 7.08 (2.64*) pg/ml. Man Whitney-U test, p=1.*

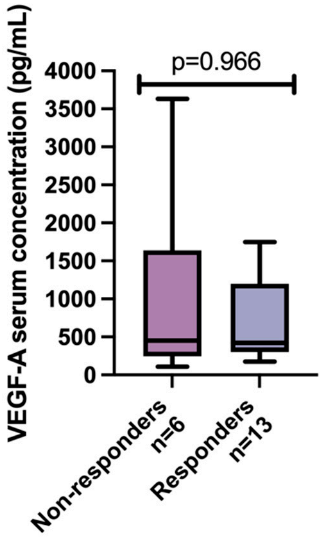

Figure S2. VEGF-A serum concentrations between non-responders and responders.

The bars represent the median with interquartile range (1st and 3rd quartile). VEGF-A serum concentration in non-responders' group: 450.552 (1393.43) *pg/ml and responders' group:* 422.674 (896.94*) pg/ml. Man Whitney-U test, p=0.966.*

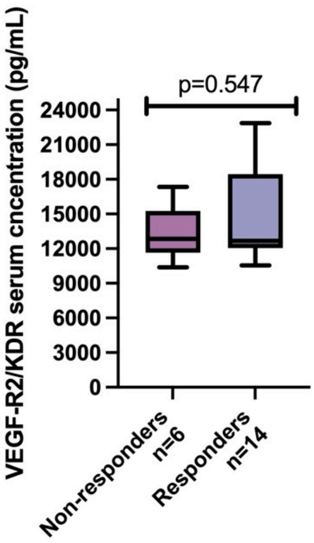

Figure S3. VEGF-R2/KDR serum concentrations between non-responders and responders.
The bars represent the median with interquartile range (1st quartile and 3rd quartile). VEGF-R2/KDR serum concentration in non-responders' group: 12845.475 (3602.8) pg/ml and responders' group: 12759.200 (6864.58*) pg/ml. Man Whitney-U test, p=0.547.*
